# A Highly Discriminative Hybrid Feature Selection Algorithm for Cancer Diagnosis

**DOI:** 10.1155/2022/1056490

**Published:** 2022-08-09

**Authors:** Tarneem Elemam, Mohamed Elshrkawey

**Affiliations:** Information Systems Department, Suez Canal University, Ismailia 41522, Egypt

## Abstract

Cancer is a deadly disease that occurs due to rapid and uncontrolled cell growth. In this article, a machine learning (ML) algorithm is proposed to diagnose different cancer diseases from big data. The algorithm comprises a two-stage hybrid feature selection. In the first stage, an overall ranker is initiated to combine the results of three filter-based feature evaluation methods, namely, chi-squared, *F*-statistic, and mutual information (MI). The features are then ordered according to this combination. In the second stage, the modified wrapper-based sequential forward selection is utilized to discover the optimal feature subset, using ML models such as support vector machine (SVM), decision tree (DT), random forest (RF), and *K*-nearest neighbor (*K*NN) classifiers. To examine the proposed algorithm, many tests have been carried out on four cancerous microarray datasets, employing in the process 10-fold cross-validation and hyperparameter tuning. The performance of the algorithm is evaluated by calculating the diagnostic accuracy. The results indicate that for the leukemia dataset, both SVM and KNN models register the highest accuracy at 100% using only 5 features. For the ovarian cancer dataset, the SVM model achieves the highest accuracy at 100% using only 6 features. For the small round blue cell tumor (SRBCT) dataset, the SVM model also achieves the highest accuracy at 100% using only 8 features. For the lung cancer dataset, the SVM model also achieves the highest accuracy at 99.57% using 19 features. By comparing with other algorithms, the results obtained from the proposed algorithm are superior in terms of the number of selected features and diagnostic accuracy.

## 1. Introduction

DNA microarray is a modern biological research technology for gene expression analysis. It has the ability to measure the expression levels of thousands of genes, during important biological operations [1]. Therefore, this technology has become an important tool, used by researchers for identifying the genes that cause cancer. In addition, it has enabled researchers to diagnose different gene-related cancer diseases [2]. As a result, numerous applications of DNA microarray technology have been implemented, which have led to the presence of a huge amount of genomic microarray data [3].

The microarray data have some specific characteristics. That is, there are a high dimensionality and a small number of samples. As such, the analysis of microarray data is considered a difficult task [4]. Since microarray data include many dimensions, causing it to be big data, dimensionality reduction (DR) is an essential preprocessing step during the classification process. The presence of many dimensions causes three main problems in the implementation of the classification task. These problems are the delay in the learning process, the increase in computational cost, and the decrease in classification accuracy [5].

DR techniques can be classified into two main approaches: feature extraction and feature selection. The feature extraction approach aims to construct the features into a new feature space with lower dimensionality. Actually, the newly constructed features are usually combinations of the original ones. Examples of feature extraction techniques include linear discriminant analysis (LDA), principal component analysis (PCA), and canonical correlation analysis (CCA). On the other hand, the feature selection approach uses the original dataset to select an optimal subset of informative features by eliminating the redundant and irrelevant features [6]. Generally, feature selection methods are categorized into four groups: filter, wrapper, embedded, and hybrid methods.

In filter methods, the most relevant features are selected through the data itself; i.e., the features are evaluated according to the intrinsic and statistical properties of the data, without using any machine learning (ML) algorithm to guide the search of relevant features [7]. Hence, these methods are distinguished by their low computational cost and scalability. Examples include information gain (IG), correlation-based feature selection (CFS), Fisher score, ReliefF, chi-squared, mutual information (MI), and minimum redundancy maximum relevance (mRMR) [8]. In wrapper methods, different feature subsets are evaluated according to the performance of a specific ML model so that the best subset is identified [9]. Although wrapper methods are more accurate than filter methods, they are more complex and slower. The most common examples of wrapper methods are forward feature selection, backward feature elimination, and recursive feature elimination, which are explained further next.

- Forward Feature Elimination. It is an iterative approach; in the beginning, there is a null model, and then, the model is fitted with each individual feature one at a time; accordingly, the feature with the highest classification accuracy is determined. Thereafter, a model is fitted with two features by trying combinations of the earlier selected feature with all other remaining features, and then, the combination of features that achieves the maximum classification accuracy is determined. This process is repeated until a subset of features outperforms all other determined subsets in terms of classification accuracy [10].
- Backward Feature Elimination. In this approach, all features are initially added to the model, and in each iteration, the least significant feature is removed based on some evaluation criteria. This process continues until no progress is detected by eliminating the features [11].
- Recursive Feature Elimination. It is an optimization algorithm and aims to find the finest feature subset. Unlike previous approaches, this approach continually produces a new model [12].

In embedded methods, ML models are used with their own built-in feature selection methods [13]. Examples of embedded methods are L1 (LASSO) regularization and decision tree (DT) [14]. In hybrid methods, the advantages of the filter and the wrapper methods are merged. The hybrid methods first use one or more filter-based methods, and then, the wrapper method is used to select the optimal feature subset [15]. In some cases, hybrid methods give better results than stand-alone ones [16]. In this article, a modified feature selection technique, which is defined as a wrapper-based sequential forward selection technique, is proposed.

In recent years, each of the ingredients of the proposed system has been the topic of much research work. As far as ML models are concerned, numerous studies have focused on employing them for cancer diagnosis. In [17], the authors present a review of 48 articles on the role of ML in disease prediction, concluding that the support vector machine (SVM) classifier is applied most frequently, followed by naive Bayes (NB). Regarding accuracy, they see that the random forest (RF) model is the best. This view of RF is shared by the authors of [18] who test five ML models, namely, SVM, DT, RF, NB, and gradient boosting (GB), to classify the samples into cancerous and noncancerous, and they report that RF achieves the best performance. The same view is also shared by the authors of [19], who use ten models for classifying cancer patients, and they report that RF with Wilcoxon signed rank-sum (WCSRS) test gives more accurate predictions than LDA, quadratic discriminant analysis (QDA), NB, Gaussian process classification (GPC), SVM, artificial neural network (ANN), logistic regression (LR), DT, and AdaBoost (AB). Another view is shared by the authors of [20], who report that SVM provides better classification based on their experiments with SVM and NB. In [21], the authors compare the performance of three ML models, namely, *K* nearest neighbors (*K*NN), SVM, and NB for the prediction of cancer among other diseases. They report that *K*NN model outperforms the other two models. In [22], the authors evaluate the performance of ML models for the purpose of biomarker prediction and report that DT yields higher performance than LDA and NB. In [23], the authors use a deep learning (DL)-based multimodel ensemble method, based on five ML models: *K*NN, SVM, DT, RF, and GB for cancer prediction. They show that the ensemble technique achieves better results than individual base models. In [24], the authors present three ML models, namely, SVM, ANN, and DT, to classify five tumor types. They report that both SVM and ANN can be used efficiently for this classification task. DT can also be used in this classification but is not efficient as well as others.

Some more relevant studies in the context of disease diagnosis using ML are in order. In [25], the authors propose an ensemble learning framework to solve positive-unlabeled learning problems in predicting miRNA-disease associations. The framework consists of a semi-supervised K-means method and a sub-aging method, combined with an effective random vector functional link network as a prediction model. In [26], the authors develop a hybrid learning framework to forecast multistep-ahead meningitis cases. The proposed framework combines signal decomposition, a weighted integrated strategy. In [27], an ML pipeline is suggested for the accurate prediction of heart disease. It includes preprocessing and entropy-based feature engineering. Performance analysis is carried out on LR, DT, RF, NB, KNN, SVM, AB, and XGBoost. In [28], the authors utilize an ensemble ML technique in hybrid integrations to predict dengue disease getting high accuracy. In [29], ML approaches such as Bayesian regression neural network, cubist regression, KNN, quantile random forest, and support vector regression are used stand-alone and coupled with variational mode decomposition for predicting COVID-19 cases.

To overcome the dimensionality problem, a set of useful feature selection methods have been proposed to analyze gene profiling for selecting the highly distinguished genes, which are called biomarkers. In [30], the authors propose a gene selection programming (GSP) method for selecting relevant genes to effectively classify cancer. SVM with a linear kernel is used as a classifier of the GSP. The proposed method is tested on ten microarray datasets. The experiments demonstrate that GSP is the most effective for removing irrelevant and redundant genes from microarray datasets. In addition, the authors demonstrate that the subset of genes selected by GSP achieves the highest classification accuracy, with the lowest processing time. In [31], the authors present a two-stage gene selection method, called mRMR-COA-HS. In the first stage, the number of genes is reduced by mRMR. In the second stage, a combination of cuckoo optimization algorithm (COA) and harmony search (HS) with the SVM classifier is used. This method is performed on four microarray datasets. The authors report that the mRMR-COA-HS method is significantly superior to other methods. In [32], the authors propose a feature selection algorithm based on relevance, redundancy, and complementarity (FS-RRC). To illustrate the performance of FS-RRC, FS-RRC is compared with eleven effective feature selection methods on fifteen public biological datasets and two synthetic datasets. The experimental results demonstrate the superiority of FS-RRC. In [33], the authors develop a novel hybrid wrapper approach called BTLBOGSA for gene selection. This approach is based on integrating the characteristics of teaching learning-based algorithm (TLBO) and gravitational search algorithm (GSA). The proposed method employs an NB classifier as a fitness function to select the extremely important genes that can help accurately to classify cancer. The effectiveness of this method is tested on ten biological datasets. Experimental results show that this method clearly outperforms other available filter and wrapper methods.

In [34], the authors propose a customized similarity measure using a fuzzy rough quick reduct algorithm for feature selection, and this method is evaluated using leukemia, lung, and ovarian cancer gene expression datasets on RF classifier. The authors conclude that the proposed method shows promising results compared with other methods. In [35], the authors present a two-stage gene selection method, called MI-GA. In the first stage, MI-based gene selection is used. In the second stage, genetic algorithm (GA)-based gene selection is used. The efficiency of the proposed method is verified using the SVM classifier, which uses five variations, and each variation uses different kernel functions. This method is performed on colon, lung, and ovarian cancer datasets. The results show that the proposed MI-GA gene selection method gives better results than the existing methods and produces maximum classification accuracy. In [36], the authors introduce a distributed feature selection (DFS) strategy using symmetric uncertainty (SU), CFS, and multilayer perceptron (MLP) through distribution across multiple clusters. Well-known classifiers are applied to the selected features. These classifiers include RIDOR, SVM, *K*NN, and simple cart (SC). The experimental implementation of this strategy accomplishes about 57% success rate and 18% competitive rate compared with traditional methods when applied to seven high-dimensional microarray datasets and one lower-dimension dataset. In [37], the authors use MapReduce (MR)-based approach to present a novel distributed method. The presented algorithm consists of MR-based Fisher score (mrFScore), MR-based ReliefF (mrReliefF), and MR-based probabilistic neural network (mrPNN) using the weighted chaotic grey wolf optimization technique (WCGWO). The authors report that the performance of WCGWO-mrPNN outperforms the other methods, when tested on seven well-known datasets that have high-dimensional microarray classification.

In [38], the Jaya optimization algorithm is exploited to introduce a novel feature selection approach called FSJaya. To evaluate the FSJaya approach efficiency, four classifiers, namely, NB, KNN, LDA, and rep tree (RT), are used on several datasets with different dimensions. The authors show that the proposed approach is efficiently able to remove the redundant features and clearly outperforms feature selection by implementing a genetic algorithm (FSGA), feature selection by applying differential evolutionary (FSDE) approaches, and feature selection by using a particle swarm optimization algorithm (FSPSO). In [39], the authors propose the G-Forest algorithm, which is tested on two datasets of two types of cancers, leukemia and diffuse large B-cell lymphoma (DLBCL). The results report that G-Forest enhances accuracy up to 14% and reduces costs up to 56% on average compared with other methods. In [40], an optimization algorithm called the elephant search algorithm (ESA) is suggested to select the best gene expressions. Firefly search (FFS) is also employed to find out the efficiency of this method in the feature selection process. In addition, a stochastic gradient descent-based deep neural network as DL with softmax activation function is used on the reduced features to improve the classification. The experiments are performed on ten common cancer microarray datasets, which are obtained from the UCI machine learning repository. The authors state that the proposed method is as important as the best method presented in the literature.

In [41], the authors present a hybrid algorithm called SARA, which is implemented by simulated annealing (SA) and Rao algorithm (RA) for selecting the optimal subset of genes and classifying cancer. The presented method consists of two stages. The first stage uses mRMR for feature preselection. While the second stage uses SARA as a wrapper method. Furthermore, the log sigmoidal function is introduced as an encoding scheme to convert the continuous version of simulated annealing-Rao algorithm (SARA) into a discrete optimization algorithm. The proposed method is implemented on three binary-class and four multi-class datasets. The authors report that this method selects the highly discriminating genes with high classification accuracy. Particularly, for small round blue cell tumor (SRBCT) dataset, it achieves high classification accuracy at 99.81% using only five informative genes. In [42], the authors propose the cuckoo search method guided by the memory-based mechanism to store the most informative features that are determined by the best solutions. The proposed algorithm is compared with the original algorithm using twelve microarray datasets. The experimental results indicate that the proposed algorithm outperforms the original and contemporary algorithms. In [43], the authors provide a feature selection method based on the artificial electric field algorithm (AEFA), called FSAEFA. The presented method is evaluated and compared with some other feature selection methods, namely, FSDE, FAGA, and FSPSO. This method is tested on ten datasets. The authors report that the proposed method is superior to other methods.

Based on the mentioned studies, it can be seen that there is no agreement on which ML model is best for predicting cancer. Obviously, this depends on several factors, such as the training dataset, applied methodology, selected features, and model parameters. The above studies also tell that no single feature selection approach is best in all circumstances. Thus, one has to experiment with the prediction situation at hand and that is what will be done in this article. In particular, extensive experiments will be conducted to determine which ML model achieves the best accuracy in predicting cancer, using the fewest number possible of features. Therefore, a brief look at each model used in this article is in order.

The SVM model is used for both classification and regression problems [44]. SVM creates a decision boundary (hyperplane) in an N-dimensional space (being N the number of features) to separate data from different classes. The main goal is to maximize the distance between this hyperplane and the data examples that are closest to it (support vectors) [45]. SVM is frequently applied in bioinformatics and medical analysis, especially for gene classification [46]. The DT model is used to create a training path to predict classes by deduction of the learning decision rules from the training dataset. It presents a simple visualization of results [47]. The RF model is categorized as an ensemble ML model, as it consists of a combination of DT models. Each DT is created by a random vector sampled independently from the input vectors, casting at the end a vote for the most likely class the input vector belongs to [48]. The KNN model is the simplest supervised ML model. It is utilized for both classification and regression predictive problems. It depends on the value of K or the number of predefined nearest neighbors. To classify the test object, the distance between neighboring objects is measured, and then, the majority class among K neighbors is assigned to the test object [49].

In this article, a new two-stage hybrid feature selection algorithm is proposed. In the first stage, a robust overall ranker is constructed to combine the results of three different filter methods, namely, chi-squared, *F*-statistic, and MI as a preprocessing stage to improve the feature selection procedure. In the second stage, the feature selection procedure is implemented using a modified wrapper-based sequential forward selection technique to select the most predictive and informative genes that can help accurately classify cancer. SVM, DT, RF, and *K*NN classifiers are utilized in the selection of the optimal feature subset. Extensive experiments are conducted on four different cancerous microarray datasets, namely, leukemia, ovarian cancer, SRBCT, and lung cancer to demonstrate the effectiveness and efficiency of the proposed method. The proposed system outperforms state-of-the-art systems in terms of the number of selected genes and classification accuracy.

The rest of the article is structured as follows.  Section 2 describes the proposed cancer prediction system.  Section 3 details the experimental conditions, results obtained, and comparisons with other state-of-the-art methods. Finally, Section 4 presents the conclusions and future work.

## 2. Materials and Methods

**Figure 1:**
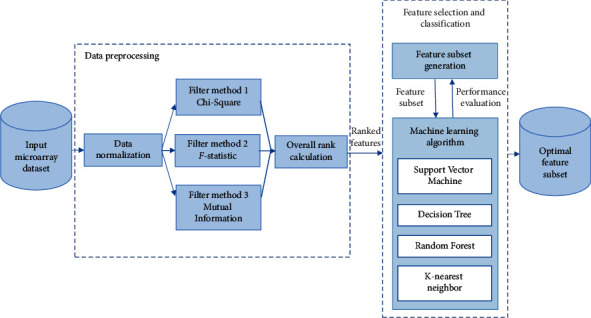
Proposed cancer prediction system.

This section presents an explanation for the conceptual structure of the proposed cancer prediction system. As shown in Figure 1, the system is composed of two successive phases: the data preprocessing phase and the phase of the feature selection and classification. In the data preprocessing phase, the feature values are normalized and the features are ranked according to their importance to make them suitable for the feature selection procedure. In the feature selection and classification phase, the models are trained and tested to identify the fewest number of features that achieve the highest accuracy. Moreover, the features that reduce the performance of ML model are excluded.

### 2.1. Data Preprocessing Phase

The data preprocessing phase is essential for cleaning the data and making it suitable for building the ML model, and this will increase the accuracy and efficiency of the model. The data preprocessing phase includes the following two processes.

#### 2.1.1. Data Normalization

Each feature value *x* of a column *X* is normalized using a min-max technique. Consequently, each feature value *x* is scaled according to the following equation to a value *x*_scaled_ ∈ [0,1]:

(1) $$\begin{array}{c} x_{\text{scaled}}=\frac{x-\text{min}\left(X\right)}{\max\left(X\right)-\text{min}\left(X\right)}, \end{array}$$

where min (*X*) and max (*X*) are the minimum and maximum values of the feature column *X*.

#### 2.1.2. Feature Ranking

The main goal of this step is to order the features according to their importance. So, filter-based feature evaluation methods are employed to evaluate the significance of each feature. In particular, three filter methods are applied: chi-squared, *F*-statistic, and MI [50].

*(i) Filter Methods*. Chi-Squared (*X*^2^): this statistic examines the dependence between two random variables, in our case a feature and the target (decision) variable. To calculate the chi-squared statistic, the first step is to create from the dataset a contingency table, having *r* rows, where *r* is the number of distinct values of the feature, and *c* columns, where *c* is the number of distinct classes of the target. At each entry *i*, *j* in the table, we place both the observed frequency and expected frequency for feature value *i* and class *j*. The observed frequency *O*_*ij*_ is the number of times value *i* appears with class *j* in the dataset. The expected frequency *E*_*ij*_ is the fraction of times value *i* appears as a value for the feature, multiplied by the number of cases of class *j*. Now, the chi-squared statistic can be computed as follows [51]:

(2) $$\begin{array}{c} X^{2}=\sum_{i=1}^{r}\sum_{j=1}^{c}\frac{{\left(O_{ij}-E_{ij}\right)}^{2}}{E_{ij}}. \end{array}$$

A zero chi-squared value means that the two variables are entirely independent.

*F*-statistic: An *F*-statistic or *F*-test is a family of statistical tests that calculates the ratio between variances. A larger *F* value means the feature is more discriminative. For a dataset of two classes, positive and negative, the *F*-statistic of the *i*th feature can be calculated using [52]the following equation:

(3) $$\begin{array}{c} F_{i}=\frac{{\left({\bar{x}}_{i}^{\left(+\right)}-{\bar{x}}_{i}\right)}^{2}+{\left({\bar{x}}_{i}^{\left(-\right)}-{\bar{x}}_{i}\right)}^{2}}{1/n_{+}-1\sum_{k=1}^{n_{+}}{\left(x_{k,i}^{\left(+\right)}-{\bar{x}}_{i}^{\left(+\right)}\right)}^{2}+1/n_{-}-1\sum_{k=1}^{n_{-}}{\left(x_{k,i}^{\left(-\right)}-{\bar{x}}_{i}^{\left(-\right)}\right)}^{2}}, \end{array}$$

where*n* is the total number of cases, *n*_+_ is the number of positive cases, *n*_−_ is the number of negative cases, $\bar{x_{i}}$ is the average of the values of the *i*th feature, ${\bar{x_{i}}}^{\left(+\right)}$ is the average of the values of the *i*th feature for the positive cases, ${\bar{x_{i}}}^{\left(-\right)}$ is the average of the values of the *i*th feature for the negative cases, *x*_*k*,*i*_^(+)^ is the value of the *i* th feature of *k* th positive case, and *x*_*k*,*i*_^(−)^ is the value of the *i* th feature of *k* th negative case. We can see in the above equation that the numerator measures how far the feature average for each class is from the feature average for the dataset as a whole, whereas the denominator is the variances of both classes. Clearly, the fraction will get bigger as the numerator gets bigger and the denominator gets smaller.

Mutual Information (MI): The mutual information, *I*(*X*; *Y*), is calculated between two random variables, *X* and *Y*, and represents the information they share, or more specifically the reduction in uncertainty for one given a known value of the other. The MI between discrete random variables, *X* and *Y*, with values over spaces *𝒳* and *𝒴*, respectively, can be calculated as [53]follows:

(4) $$\begin{array}{c} I\left(X;Y\right)=\sum_{i\in X}\sum_{j\in Y}p_{X,Y}\left(i,j\right)\;\log\frac{p_{X,Y}\left(i,j\right)}{p_{X}\left(i\right)p_{Y}\left(j\right)}, \end{array}$$

where *p*_*X*,*Y*_(*i*, *j*) is the joint probability distribution of *X* and *Y*, and *p*_*X*_(*i*) and *p*_*Y*_(*j*) are the marginal probability distributions of *X* and *Y*, respectively. If the log is taken to the base 2, the units are bits. A zero MI means that the variables are completely unrelated, which is because if *X* and *Y* are independent, then *p*_*X*,*Y*_(*i*, *j*) = *p*_*X*_(*i*)*p*_*Y*_(*j*) so that *p*_*X*,*Y*_(*i*, *j*)/*p*_*X*_(*i*)*p*_*Y*_(*j*) = 1, whose log is 0.

**Figure 2:**
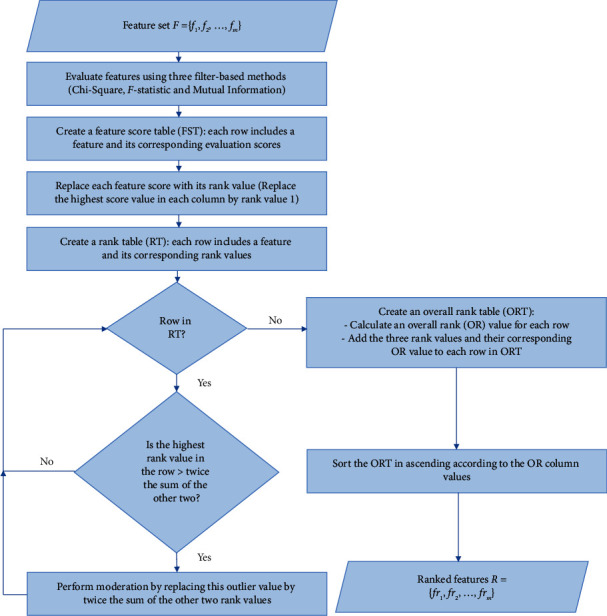
Feature ranking process.

*(ii) Overall Ranking Algorithm.* According to the proposed work, the feature ranking process is performed based on gathering the separated results of the mentioned filters together. The complete feature ranking process is shown in Figure 2, which is carried out through five detailed steps as follows.

(1) A feature score table (FST) of *m* rows and 4 columns is constructed, where *m* is the number of dataset features. The first column is assigned to feature names and the next three columns are assigned to their evaluation values by the three filters: chi-squared, *F*-statistic, and MI.
(2) A rank table (RT) with *m* rows and 4 columns is created. The first column for the feature names is assigned. Each value in the next three columns of the RT is deduced from its corresponding value in the FST as follows:
    The score value of each feature in the FST is replaced by a corresponding rank value in RT.
    The value 1 represents the highest rank and is assigned to the feature with the highest score in each of the filter columns in the FST.
    The rank value is increased by 1 for the feature score, which is directly below the previous score in each of the filter columns in the FST.
    The previous step is repeated until reaching the lowest rank with value *m*.
(3) In the RT, the outliers (extreme) of the rank values are detected as follows:
    In the row of each feature, the highest rank value of the three filters is examined.
    If the highest rank value is less than or equal to twice the sum of the other two, then all rank values will remain the same.
    Otherwise, if one of the rank values is greater than twice the sum of the other two, then it means that there is an outlier and it needs moderation.
    The required moderation is performed by replacing that outlier value with twice the sum of the other rank values.
    For example, if the row of some feature in the RT is [8,2,1], then the 8 will be considered an outlier, since 8 > 6. Thus, the row will be modified to [6,2,1].
(4) An overall rank table (ORT) with 5 columns is constructed, and the first column is filled with feature names. Next, the following procedures are performed:
    The next three columns are filled with the rank values of the 3 filters after moderation.
    For each feature, the overall rank (OR) value is deduced by summing the three rank values of the feature's row to be a single value in the fifth column.
(5) Ascendingly, the ORT is sorted using the OR values of the fifth column as a key. The features will be ordered from the most important, at the top, to the least important at the bottom.

Algorithm 1 illustrates the pseudo-code of the overall ranking algorithm.

### 2.2. Feature Selection and Classification Phase

In this section, the two processes of feature selection and classification are explained.

#### 2.2.1. Feature Selection

Feature selection is a very crucial step because the inclusion of inconsequential and redundant features negatively affects the model performance significantly. By selecting relevant features from the raw dataset, the learning model is improved in many ways: (i) avoiding learning from noise and overﬁtting, (ii) improving accuracy, and (iii) reducing training time. In addition, working with more informative features contributes to early diagnosis. As mentioned in Section 1, there are four types of feature selection methods, namely, filter-based, wrapper-based, embedded-based, and hybrid-based methods.

In this article, a modified wrapper-based sequential forward selection technique is presented. In this model, the selection technique starts by adding the highest overall rank feature to an empty subset and then it measures the model's performance. Next, a set of successive iterations are performed. In each iteration, only one feature is added to the subset and performance is measured. If the newly added feature improves the performance of the model, it will remain within the subset. Otherwise, the added feature will be removed. Likewise, the remaining features are added and evaluated one by one to the features kept in the subset. In the last iteration, the features that are kept in the subset are the features that optimize the classification accuracy.

#### 2.2.2. Classification

The classification technique is applied to categorize data into a set of classes using supervised ML techniques. There are a variety of classification techniques for classifying microarray datasets. Based on the recent literature on cancer prediction (as summarized in Section 1), the present work implements four prediction models, namely, SVM, DT, RF, and *K*NN.

To optimize and refine the performance of the proposed models, the hyperparameter tuning technique is implemented to pass various parameters into the model using the grid search method that takes a set of possible values for each hyperparameter, evaluates the performance for each combination of them, and in the end selects the combination, which achieves the best performance.

The *k*-fold cross-validation approach is also utilized to get the best performance for the models. In the present work, *k*=10 is used, so the dataset is split into 10-fold of approximately the same size. Then, ninefolds are utilized for training and only onefold for the testing. This process is repeated until each of the 10-fold has been used as a testing set to ensure that each case in the dataset has been classified by the model. For each fold, the performance of the model is calculated, and eventually, the average performance is obtained from the 10-fold.

In addition, the accuracy is used as a vital metric for evaluating the performance of ML models. The accuracy is deduced as follows:

(5) $$\begin{array}{c} \text{Accuracy}=\frac{TP+TN}{TP+TN+FP+FN}, \end{array}$$

where *TP* (true positive) is the number of cases belonging to the class and correctly labeled as such, *FP* (false positive) is the number of cases belonging to the class but incorrectly labeled as not, *TN* (true negative) is the number of cases not belonging to the class and correctly labeled as such, and *FN* (false negative) is the number of cases belonging to the class but incorrectly labeled as not.

**Figure 3:**
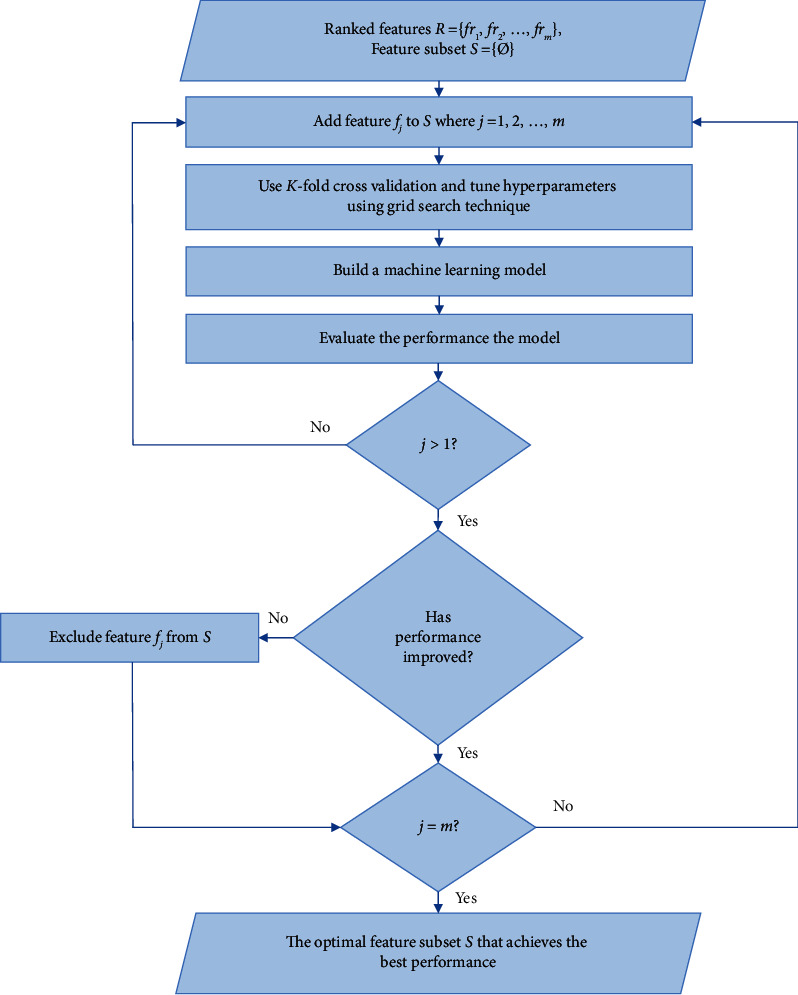
Feature selection and classification process.

*(i) Feature Selection and Classification Algorithm.* After ordering the features from the most significant to the least based on OR values in the ORT, the feature selection procedure is performed. The complete feature selection and classification process are shown in Figure 3, which is carried out through the following steps.

(1) The most important feature that is in the first row of the ORT to an empty feature subset is added.
(2) 10-fold cross-validation is used for the feature subset and tune hyperparameters using the grid search technique.
(3) An ML model is built using this subset of features.
(4) The accuracy of the ML model is calculated, and it is called as the previous accuracy.
(5) The next feature that is in the next row of the ORT to the feature subset is appended.
(6) 10-fold cross-validation is used for the feature subset and tune hyperparameters using the grid search technique.
(7) An ML model is built using this subset of features.
(8) The accuracy of the ML model is calculated, and it is called as the current accuracy.
(9) The current accuracy is compared with the previous accuracy as follows:
    If the current accuracy is less than or equal to the previous accuracy, then the last added feature is excluded from the feature subset.
    Otherwise, if the current accuracy is greater than the previous accuracy, then the previous accuracy is made equal to the current accuracy.
(10) The steps starting from step 5 are repeated until reaching the end of the ORT.
(11) The optimum feature subset and its accuracy (the previous accuracy) are returned.

Algorithm 2 illustrates the pseudo-code of the feature selection and the classification process.

## 3. Results and Discussion

The proposed system was tested by performing extensive experiments on four publicly available microarray datasets [54] shown in Table 1. The system, based on Apache Spark, was written in Python. Some API libraries that are integrated with Spark were used such as Spark's MLlib to implement the feature selection and classification algorithm. Python libraries were used to implement the feature ranking algorithm. The proposed system was implemented on a Spark cluster, which consists of one master node and two slave nodes. Every node was deployed with the same physical environment, i.e., Intel (R) Core (TM) i7-4510 U CPU @ 2.00 GHz, 2.60 GHz, and 8 GB memory.

It should be noted that Spark provides an interface for programming entire clusters with implicit data parallelism and fault tolerance. It can work with structured data such as CSV files and unstructured data such as JSON files [55]. Spark provides high-level APIs in Scala, Java, Python, and R for libraries such as MLlib (Machine Learning Library) for ML, Spark Streaming for stream processing, GraphX for graph analysis, and Spark SQL for structured data processing [56]. MLlib implements ML prediction models, hyperparameter tuning, and cross-validation. It is divided into two main packages: spark.mllib and spark.ml. spark.mllib is built on top of RDDs, and spark.ml is built on top of DataFrames. Both packages come with a variety of common ML tasks such as featurization, transformations, model training, model evaluation, and optimization. In the present work, we use the spark.ml package because it provides the pipeline API for building, debugging, and tuning ML pipelines, whereas spark.mllib includes packages for linear algebra, statistics, and other basic utilities for ML. DataFrames can automatically distinguish between numerical and categorical features and can also automatically optimize both storage and computation [57].

The methods outlined in Section 2 were followed to build the model. So, first, the filter-based feature evaluation methods were used to order the features according to their importance, and then, the ML models were trained and tested. It ends up selecting the model with the highest performing, which used the fewest number of features obtained through the modified wrapper-based sequential forward selection technique. The results of feature ranking, feature selection, and classification process are described in this section, in addition to presenting a comparison of the performance of the proposed method in terms of the number of selected features and classification accuracy with twelve other methods.

### 3.1. Feature Ranking Results

Tables 2 and 3 display the metric scores obtained for only twenty features of two microarray datasets: leukemia and ovarian cancer, respectively. Scores were obtained by the three metrics, chi-squared, *F*-statistic, and MI, applying equations (2), (3), and (4), respectively. It can be observed that the same feature is ranked differently by each metric. For example, for the leukemia dataset as shown in Table 2, the chi-squared statistic method sees “M27891_at” as the most important feature, and this view is not shared by the *F*-statistic and MI. Actually, *F*-statistic sees “X95735_at” as the most important feature, but MI sees “M23197_at” as the most important. For the ovarian dataset, as shown in Table 3, both chi-squared statistic method and *F*-statistic see “MZ245.24466” as the most important feature, while MI sees “MZ244.95245” as the most important feature, likewise for both SRBCT and lung cancer datasets. For this variation, an approach described in Section 2 will be used to find an overall rank for each feature based on the collective view of the three metrics.

For the feature ranking process, after creating a feature score table (FST) for each dataset, a rank table (RT) is created for each of them as shown in Tables 4 and 5. These tables show the rank of only twenty features of leukemia and ovarian cancer datasets, respectively. Here, each metric value is replaced by its rank among its peers. For leukemia and ovarian cancer datasets, the results of the overall rank of the top twenty features—after moderating the outliers—are shown in Tables 6 and 7, respectively. In these tables, a moderated outlier is set in bold, likewise for both SRBCT and lung cancer datasets.

### 3.2. Feature Selection and Classification Results

Four ML models were explored for cancer prediction, namely, SVM, DT, RF, and *K*NN. These models in particular are chosen based on reviewing the recent research on cancer prediction as summarized in Section 1. For each dataset, to evaluate the performance of the four candidate ML models, some experiments were carried out, one using all features and the others using the features ranked by their overall rank (the proposed approach) to determine which feature subset achieves the best accuracy. 10-fold cross-validation is used to evaluate each of the four ML models. This means that—of all cases of the dataset—90% were used for training and 10% for testing. From the test results, for each model and for each fold, the accuracy metric was calculated using equation (5). The results of accuracy metric were then averaged for all the 10-fold. The average of the accuracy was then taken to give a single number for each model indicating its performance. The performance of the models using full features and the features selected by the proposed wrapper method is presented in the following subsections.

#### 3.2.1. Performance of the Models Using All Features

In this experiment, for each dataset, the performance of the four ML models when trained and tested on all features was measured.

As can be seen in Table 8, for the leukemia dataset, both RF and SVM models achieve the best average accuracy at 98.57%. For the ovarian cancer dataset, the SVM model outperforms the other models by achieving the highest average accuracy at 100%. For the SRBCT dataset, both RF and SVM models register the best average accuracy at 100%. While for the lung cancer dataset, the RF model achieves the best average accuracy at 99.57%.

#### 3.2.2. Performance of the Models Using the Features Selected by the Proposed Wrapper-Based Sequential Forward Selection Method

In this experiment, for each dataset, the performance for the four ML models was measured when they were trained and tested on subsets of features that were selected according to the proposed wrapper method.

For each dataset, the best subset of features that achieves the best accuracy is shown in Table 9.

As can be seen in Table 8, for the leukemia dataset, both SVM and *K*NN models register the best average accuracy at 100% using only 5 features. For the ovarian cancer dataset, the SVM model outperforms the other models by achieving the best average accuracy at 100% using only 6 features. For the SRBCT dataset, the SVM model also outperforms the other models by achieving the best average accuracy at 100% using only 8 features. While for the lung cancer dataset, the SVM model achieves the highest average accuracy at 99.57% using 19 features.

### 3.3. Comparison with Other Algorithms

Table 10 reports the comparative results of the four microarray datasets introduced above. In particular, the results of the proposed algorithm are compared with those of twelve algorithms in the literature. From the comparison, it can be easily realized that the proposed algorithm is promising in terms of classification accuracy and number of selected features for all used datasets. In particular, an accuracy of at least 99.57% is obtained throughout.

## 4. Conclusions

This article presents a robust machine learning (ML)-based algorithm to diagnose different cancer diseases using microarray datasets. The algorithm can effectively eliminate irrelevant and redundant genes. The output of the algorithm has high stability and classification accuracy. When the results of the algorithm are compared with those of similar algorithms, the proposed algorithm showed clear superiority. In particular, it selected a smaller number of genes and yielded a higher level of accuracy. Furthermore, the time and storage cost of the algorithm are very appealing, making it optimal for big data.

An interesting future extension would be to adapt and verify the proposed algorithm on more realistic and benchmark microarray datasets of bigger sizes. Also, implementation using Hadoop/MapReduce platforms could be explored. In particular, to make the algorithm faster and more efficient when dealing with high-dimensional data, we intend to develop a parallel version to be run on cluster/cloud computing facilities.

## Figures and Tables

**Algorithm 1:**
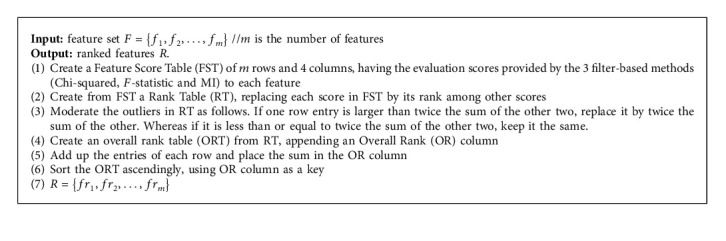
Overall ranking.

**Algorithm 2:**
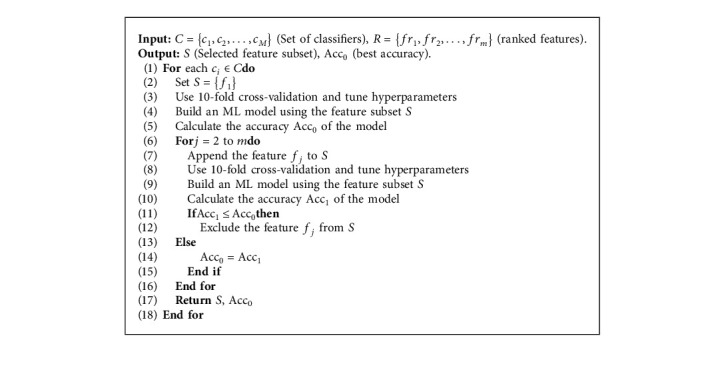
Feature selection and classification process.

**Table 1:** Dataset description.

Dataset	Number of samples	Number of features	Number of classes	Notes
Leukemia	72	7129	2 (binary class)	ALL: 47, AML: 25
Ovarian cancer	253	15154	2 (binary class)	Cancer: 162, normal: 91
SRBCT	83	2308	4 (multi-class)	EWS: 29, BL: 11, NB: 18, RMS: 25
Lung cancer	203	12600	5 (multi-class)	AD: 139, NL: 17, SMCL: 6, SQ: 21, COID: 20

ALL—acute lymphocytic leukemia; AML—acute myeloid leukemia; EWS—Ewing's sarcoma; BL—Burkitt's lymphoma; NB—neuroblastoma; RMS—rhabdomyosarcoma; AD—adenocarcinoma; NL—normal lung; SMCL—small cell lung cancer; SQ—squamous cell carcinoma; COID—carcinoid.

**Table 2:** Feature score table (FST) for the leukemia dataset.

Feature	*X* ^2^	*F*-Statistic	MI
D88422_at	10.9959	36.676	0.442189
M11722_at	6.34719	34.1799	0.411207
M16038_at	8.98639	68.645	0.342855
M19507_at	10.7386	45.239	0.323609
M22960_at	6.23369	65.0347	0.304583
M23197_at	9.65571	80.6443	0.524171
M27891_at	14.4819	69.3333	0.490489
M63138_at	7.87681	64.6046	0.35717
M84526_at	9.52866	73.2956	0.429966
M92287_at	5.66233	43.1572	0.385647
M96326_rna1_at	7.75604	42.5043	0.364215
U05259_rna1_at	5.78902	38.5285	0.335938
U46499_at	11.0199	69.8495	0.418786
X17042_at	10.1589	81.3535	0.352082
X59417_at	4.90528	45.0031	0.344774
X61587_at	4.87439	55.0604	0.330945
X62654_rna1_at	4.60257	43.7079	0.394887
X95735_at	8.57247	119.315	0.497266
L09209_s_at	8.18643	71.1058	0.465179
M31523_at	4.76099	41.6929	0.478039

**Table 3:** Feature score table (FST) for the ovarian cancer dataset.

Feature	*X* ^2^	*F*-Statistic	MI
MZ244.36855	31.2944	358.634	0.40996
MZ244.66041	31.4802	642.088	0.517489
MZ244.95245	35.4655	857.449	0.5695
MZ245.24466	35.8013	905.675	0.552878
MZ245.53704	33.7166	833.195	0.541194
MZ245.8296	30.9072	713.093	0.53736
MZ246.12233	28.6963	597.697	0.532594
MZ246.41524	26.7876	507.936	0.490768
MZ246.70832	25.0276	444.715	0.482529
MZ247.00158	23.932	399.491	0.470867
MZ247.295	24.0877	361.301	0.453271
MZ247.58861	23.5078	302.861	0.432823
MZ247.88239	22.6694	289.701	0.425722
MZ261.88643	13.5449	418.438	0.460344
MZ417.73207	14.4542	411.356	0.46087
MZ434.68588	11.8603	384.315	0.475778
MZ435.07512	12.2497	405.504	0.521955
MZ435.46452	12.3376	381.56	0.526363
MZ463.95962	18.2664	314.626	0.393138
MZ464.36174	18.1024	320.451	0.422442

**Table 4:** Feature rank table (RT) for the leukemia dataset. Evaluation scores are converted to ranks, with 1 being the highest rank.

Feature	*X* ^2^	*F*-Statistic	MI
D88422_at	4	41	6
M11722_at	21	51	9
M16038_at	9	8	23
M19507_at	5	20	29
M22960_at	23	9	37
M23197_at	7	3	1
M27891_at	1	7	3
M63138_at	12	10	17
M84526_at	8	4	7
M92287_at	30	26	12
M96326_rna1_at	14	28	16
U05259_rna1_at	28	37	24
U46499_at	3	6	8
X17042_at	6	2	19
X59417_at	43	21	22
X61587_at	44	14	25
X62654_rna1_at	55	24	10
X95735_at	10	1	2
L09209_s_at	11	5	5
M31523_at	49	32	4

**Table 5:** Feature rank table (RT) for the ovarian cancer dataset. Evaluation scores are converted to ranks, with 1 being the highest rank.

Feature	*X* ^2^	*F*-Statistic	MI
MZ244.36855	5	18	25
MZ244.66041	4	5	8
MZ244.95245	2	2	1
MZ245.24466	1	1	2
MZ245.53704	3	3	3
MZ245.8296	6	4	4
MZ246.12233	7	6	5
MZ246.41524	8	7	10
MZ246.70832	9	8	11
MZ247.00158	11	12	13
MZ247.295	10	17	16
MZ247.58861	12	25	18
MZ247.88239	13	29	20
MZ261.88643	58	9	15
MZ417.73207	38	10	14
MZ434.68588	110	13	12
MZ435.07512	91	11	7
MZ435.46452	89	14	6
MZ463.95962	18	24	30
MZ464.36174	19	21	22

**Table 6:** Overall rank table (ORT) for the leukemia dataset. Features are arranged from the most important, at the top, to the least important at the bottom according to their assigned overall rank (OR), which is calculated after moderating the outliers (shown in bold) as per Algorithm 1. The smaller the overall rank, the more significant the feature.

Feature	*X* ^2^	*F*-Statistic	MI	Overall rank (OR)
X95735_at	**6**	1	2	9
M23197_at	7	3	1	11
M27891_at	1	7	3	11
U46499_at	3	6	8	17
M84526_at	8	4	7	19
L09209_s_at	11	5	5	21
X17042_at	6	2	**16**	24
D88422_at	4	**20**	6	30
M63138_at	12	10	17	39
M16038_at	9	8	23	40
M19507_at	5	20	29	54
M96326_rna1_at	14	28	16	58
M92287_at	30	26	12	68
M22960_at	23	9	37	69
M11722_at	21	51	9	81
X61587_at	44	14	25	83
M31523_at	49	32	4	85
X59417_at	43	21	22	86
U05259_rna1_at	28	37	24	89
X62654_rna1_at	55	24	10	89

**Table 7:** Overall rank table (ORT) for the ovarian cancer dataset. Features are arranged from the most important, at the top, to the least important at the bottom according to their assigned overall rank (OR), which is calculated after moderating the outliers (shown in bold) as per Algorithm 1. The smaller the overall rank, the more significant the feature.

Feature	*X* ^2^	*F*-Statistic	MI	Overall rank (OR)
MZ245.24466	1	1	2	4
MZ244.95245	2	2	1	5
MZ245.53704	3	3	3	9
MZ245.8296	6	4	4	14
MZ244.66041	4	5	8	17
MZ246.12233	7	6	5	18
MZ246.41524	8	7	10	25
MZ246.70832	9	8	11	28
MZ247.00158	11	12	13	36
MZ247.295	10	17	16	43
MZ244.36855	5	18	25	48
MZ435.07512	**36**	11	7	54
MZ247.58861	12	25	18	55
MZ435.46452	**40**	14	6	60
MZ464.36174	19	21	22	62
MZ417.73207	38	10	14	62
MZ247.88239	13	29	20	62
MZ463.95962	18	24	30	72
MZ261.88643	**48**	9	15	72
MZ434.68588	**50**	13	12	75

**Table 8:** Average classification accuracy using four classifiers on four biological datasets. The number of features is shown in parentheses. The best results are shown in bold font.

Dataset	Classifier	Accuracy using full features (%)	Accuracy using selected features (%)
Leukemia (7129)	SVM	**98.57**	**100 (5)**
	DT	85.89	98.57 (3)
	RF	**98.57**	98.57 (3)
	*K*NN	92.14	**100 (5)**

Ovarian cancer (15154)	SVM	**100**	**100 (6)**
	DT	97.60	98.80 (4)
	RF	99.60	99.60 (10)
	*K*NN	95.28	100 (10)

SRBCT (2308)	SVM	**100**	**100 (8)**
	DT	83.19	96.67 (8)
	RF	**100**	98.75 (8)
	*K*NN	88.19	100 (10)

Lung cancer (12600)	SVM	99.42	**99.57 (19)**
	DT	97.85	99.14 (18)
	RF	**99.57**	99.43 (20)
	*K*NN	94.99	99.57 (22)

**Table 9:** Listing of best subset of features that achieves the best accuracy.

Dataset	Selected features
Leukemia	HG1612-HT1612_at, M23197_at, M27891_at, X17042_at, X95735_at
Ovarian cancer	MZ221.86191, MZ244.36855, MZ244.95245, MZ245.24466, MZ435.07512, MZ464.76404
SRBCT	gene123, gene153, gene187, gene509, gene742, gene1389, gene1601, gene1955
Lung cancer	38138_at, 38239_at, 35622_at, 36894_at, 37545_at, 40093_at, 34842_at, 36119_at, 36160_s_at, 37302_at, 37305_at, 38032_at, 38065_at, 40193_at, 40619_at, 41289_at, 32542_at, 1814_at, 893_at

**Table 10:** Comparison of proposed method with some existing research: with reduced features shown inside the parentheses. The symbol “—” indicates that no information is available. The best results are shown in bold font.

Author	Algorithm	Dataset			
		Leukemia	Ovarian cancer	SRBCT	Lung cancer
[34]	Customized similarity measure using a fuzzy rough quick reduct algorithm	97.22 (7)	99.60 (9)	—	—
[35]	MI-GA	—	99.21 (20)	—	81.37 (10)
[36]	DFS strategy	98.61 (85)	—	100 (133)	—
[37]	ReliefF-WCGWO-mrPNN	89.33 (150)	99.21(200)	—	—
	Fisher score-WCGWO-mrPNN	99.21 (40)	100 (150)	—	—
[38]	FSJaya	96.74 (3531)	—	—	—
[39]	G-Forest	100 (1282)	—	—	—
[40]	ESA-DL	—	99.21 (384)	83.14 (306)	94.10 (4545)
	FFS-DL	—	97.24 (35)	93.98 (768)	93.11 (5304)
[41]	SARA	97.65 (7)	99.15 (6)	99.81 (5)	90.22 (5)
[42]	Cuckoo search	100 (650)	—	—	—
[43]	FSAEFA	96.14 (3530)	—	—	—
	Proposed method (ours)	**100 (5)**	**100 (6)**	**100 (8)**	**99.57 (19)**

## Data Availability

The microarray datasets used to support the findings of this study can be accessed at https://csse.szu.edu.cn/staff/zhuzx/Datasets.html.

## References

[1] Dabba A., Tari A., Meftali S., Mokhtari R. (2021). Gene selection and classification of microarray data method based on mutual information and moth flame algorithm. *Expert Systems with Applications*.

[2] Haznedar B., Arslan M. T., Kalinli A. (2021). Optimizing ANFIS using simulated annealing algorithm for classification of microarray gene expression cancer data. *Medical, and Biological Engineering and Computing*.

[3] Zheng X., Zhang C. (2021). Gene selection for microarray data classification via dual latent representation learning. *Neurocomputing*.

[4] Sun M., Liu K., Wu Q., Hong Q., Wang B., Zhang H. (2019). A novel ECOC algorithm for multiclass microarray data classification based on data complexity analysis. *Pattern Recognition*.

[5] Rani M. J., Karuppasamy M., Prabha M. (2021). Bacterial foraging optimization algorithm based feature selection for microarray data classification. *Materials Today Proceedings*.

[6] Lai C.-M., Huang H.-P. (2021). A gene selection algorithm using simplified swarm optimization with multi-filter ensemble technique. *Applied Soft Computing*.

[7] Solorio-Fernández S., Carrasco-Ochoa J. A., Martínez-Trinidad J. F. (2020). A review of unsupervised feature selection methods. *Artificial Intelligence Review*.

[8] Pashaei E., Pashaei E. (2022). An efficient binary chimp optimization algorithm for feature selection in biomedical data classification. *Neural Computing and Applications*.

[9] Sazzed S. (2022). Feature selection in gene expression profile employing relevancy and redundancy measures and binary whale optimization algorithm (BWOA). *Advanced Data Mining and Applications*.

[10] Nithya B., Ilango V. (2019). Evaluation of machine learning based optimized feature selection approaches and classification methods for cervical cancer prediction. *SN Applied Sciences*.

[11] Saha S., Soliman A., Rajasekaran S. (2021). A robust and stable gene selection algorithm based on graph theory and machine learning. *Human Genomics*.

[12] Balabaeva K., Kovalchuk S. (2021). Comparison of efficiency, stability and interpretability of feature selection methods for multiclassification task on medical tabular data. *International Conference on Computational Science*.

[13] Abiodun E. O., Alabdulatif A., Abiodun O. I., Alawida M., Alabdulatif A., Alkhawaldeh R. S. (2021). A systematic review of emerging feature selection optimization methods for optimal text classification: the present state and prospective opportunities. *Neural Computing and Applications*.

[14] Koul N., Manvi S. S. (2020). Feature selection from gene expression data using SVMRFE and feed-forward neural network classifier. *Advances in Communication, Signal Processing, VLSI, and Embedded Systems, *.

[15] Houssein E. H., Hassan H. N., Al-Sayed M. M., Nabil E. (2022). Gene selection for microarray cancer classification based on manta rays foraging optimization and support vector machines. *Arabian Journal for Science and Engineering*.

[16] Anter A. M., Ali M. (2020). Feature selection strategy based on hybrid crow search optimization algorithm integrated with chaos theory and fuzzy c-means algorithm for medical diagnosis problems. *Soft Computing*.

[17] Uddin S., Khan A., Hossain M. E., Moni M. A. (2019). Comparing different supervised machine learning algorithms for disease prediction. *BMC Medical Informatics and Decision Making*.

[18] Merry K. P., Tanchak K. (2020). Typecasting of microarray data using machine learning algorithms. *Procedia Computer Science*.

[19] Maniruzzaman M., Rahman M., Ahammed B. (2019). Statistical characterization and classification of colon microarray gene expression data using multiple machine learning paradigms. *Computer Methods and Programs in Biomedicine*.

[20] Shukla A. K., Singh P., Vardhan M. (2019). A new hybrid wrapper TLBO and SA with SVM approach for gene expression data. *Information Sciences*.

[21] Zhang G., Hou J., Wang J., Yan C., Luo J. (2020). Feature selection for microarray data classification using hybrid information gain and a modified binary krill herd algorithm. *Interdisciplinary Sciences: Computational Life Sciences*.

[22] Mascini N. E., Teunissen J., Noorlag R., Willems S. M., Heeren R. M. A. (2018). Tumor classification with MALDI-MSI data of tissue microarrays: a case study. *Methods*.

[23] Xiao Y., Wu J., Lin Z., Zhao X. (2018). A deep learning-based multi-model ensemble method for cancer prediction. *Computer Methods and Programs in Biomedicine*.

[24] Tuncal K., Ozkan C. Tumor classification using gene expression and machine learning models.

[25] Wu Y., Zhu D., Wang X., Zhang S. (2021). An ensemble learning framework for potential miRNA-disease association prediction with positive-unlabeled data. *Computational Biology and Chemistry*.

[26] Ribeiro M. H. D. M., Mariani V. C., Coelho L. D. S. (2020). Multi-step ahead meningitis case forecasting based on decomposition and multi-objective optimization methods. *Journal of Biomedical Informatics*.

[27] Rajendran R., Karthi A. (2022). Heart disease prediction using entropy based feature engineering and ensembling of machine learning classifiers. *Expert Systems with Applications*.

[28] Gangula R., Thirupathi L., Parupati R., Sreeveda K., Gattoju S. (2021). Ensemble machine learning based prediction of dengue disease with performance and accuracy elevation patterns. *Materials Today Proceedings*.

[29] Da Silva R. G., Ribeiro M. H. D. M., Mariani V. C., Coelho L. D. S. (2020). Forecasting Brazilian and American COVID-19 cases based on artificial intelligence coupled with climatic exogenous variables. *Chaos, Solitons and Fractals*.

[30] Alanni R., Hou J., Azzawi H., Xiang Y. (2019). A novel gene selection algorithm for cancer classification using microarray datasets. *BMC Medical Genomics*.

[31] Elyasigomari V., Lee D. A., Screen H. R. C., Shaheed M. H. (2017). Development of a two-stage gene selection method that incorporates a novel hybrid approach using the cuckoo optimization algorithm and harmony search for cancer classification. *Journal of Biomedical Informatics*.

[32] Li C., Luo X., Qi Y., Gao Z., Lin X. (2020). A new feature selection algorithm based on relevance, redundancy and complementarity. *Computers in Biology and Medicine*.

[33] Shukla A. K., Singh P., Vardhan M. (2020). Gene selection for cancer types classification using novel hybrid metaheuristics approach. *Swarm and Evolutionary Computation*.

[34] Arunkumar C., Ramakrishnan S. (2018). Attribute selection using fuzzy roughset based customized similarity measure for lung cancer microarray gene expression data. *Future Computing and Informatics Journal*.

[35] Jansi Rani M., Devaraj D. (2019). Two-stage hybrid gene selection using mutual information and genetic algorithm for cancer data classification. *Journal of Medical Systems*.

[36] Potharaju S. P., Sreedevi M. (2019). Distributed feature selection (DFS) strategy for microarray gene expression data to improve the classification performance. *Clinical Epidemiology and Global Health*.

[37] Baliarsingh S. K., Vipsita S., Gandomi A. H., Panda A., Bakshi S., Ramasubbareddy S. (2020). Analysis of high-dimensional genomic data using MapReduce based probabilistic neural network. *Computer Methods and Programs in Biomedicine*.

[38] Das H., Naik B., Behera H. S. (2020). A Jaya algorithm based wrapper method for optimal feature selection in supervised classification. *Journal of King Saud University-Computer and Information Sciences*.

[39] Abdulla M., Khasawneh M. T. (2020). G-Forest: an ensemble method for cost-sensitive feature selection in gene expression microarrays. *Artificial Intelligence in Medicine*.

[40] Panda M. (2020). Elephant search optimization combined with deep neural network for microarray data analysis. *Journal of King Saud University-Computer and Information Sciences*.

[41] Baliarsingh S. K., Muhammad K., Bakshi S. (2021). SARA: a memetic algorithm for high-dimensional biomedical data. *Applied Soft Computing*.

[42] Alzaqebah M., Briki K., Alrefai N. (2021). Memory based cuckoo search algorithm for feature selection of gene expression dataset. *Informatics in Medicine Unlocked*.

[43] Das H., Naik B., Behera H. S. (2021). Optimal selection of features using artificial electric field algorithm for classification. *Arabian Journal for Science and Engineering*.

[44] Ali L., Wajahat I., Amiri Golilarz N., Keshtkar F., Bukhari S. A. C. (2021). LDA–GA–SVM: improved hepatocellular carcinoma prediction through dimensionality reduction and genetically optimized support vector machine. *Neural Computing and Applications*.

[45] Magalhaes C., Tavares J. M. R. S., Mendes J., Vardasca R. (2021). Comparison of machine learning strategies for infrared thermography of skin cancer. *Biomedical Signal Processing and Control*.

[46] Gopal V. N., Al-Turjman F., Kumar R., Anand L., Rajesh M. (2021). Feature selection and classification in breast cancer prediction using IoT and machine learning. *Measurement*.

[47] Azhari M., Abarda A., Ettaki B., Zerouaoui J., Dakkon M. (2020). Higgs boson discovery using machine learning methods with pyspark. *Procedia Computer Science*.

[48] Bei Z., Yu Z., Luo N., Jiang C., Xu C., Feng S. (2018). Configuring in-memory cluster computing using random forest. *Future Generation Computer Systems*.

[49] Qureshi M. B., Qureshi M. B., Afzaal M., Qureshi M. S., Fayaz M. (2021). Machine learning-based EEG signals classification model for epileptic seizure detection. *Multimedia Tools and Applications*.

[50] Susan S., Hanmandlu M. (2019). Smaller feature subset selection for real-world datasets using a new mutual information with Gaussian gain. *Multidimensional Systems and Signal Processing*.

[51] Luna-Romera J. M., Martínez-Ballesteros M., García-Gutiérrez J., Riquelme J. C. (2019). External clustering validity index based on chi-squared statistical test. *Information Sciences*.

[52] Wang D., Zhang Z., Bai R., Mao Y. (2018). A hybrid system with filter approach and multiple population genetic algorithm for feature selection in credit scoring. *Journal of Computational and Applied Mathematics*.

[53] Liu Q., Xiao J., Zhu H. (2019). Feature selection for software effort estimation with localized neighborhood mutual information. *Cluster Computing*.

[54] Zhu Z., Ong Y.-S., Dash M. (2007). Markov blanket-embedded genetic algorithm for gene selection. *Pattern Recognition*.

[55] Ed-daoudy A., Maalmi K. (2019). A new internet of things architecture for real-time prediction of various diseases using machine learning on big data environment. *Journal of Big Data*.

[56] Fu J., Sun J., Wang K. Spark–a big data processing platform for machine learning.

[57] Salloum S., Dautov R., Chen X., Peng P. X., Huang J. Z. (2016). Big data analytics on Apache Spark. *International Journal of Data Science and Analytics*.

